# Exchange of Cytosolic Content between T Cells and Tumor Cells Activates CD4 T Cells and Impedes Cancer Growth 

**DOI:** 10.1371/journal.pone.0078558

**Published:** 2013-10-24

**Authors:** Matthias Hardtke-Wolenski, Lilli Kraus, Christel Schmetz, Britta Trautewig, Fatih Noyan, Florian W. R. Vondran, Hueseyin Bektas, Juergen Klempnauer, Elmar Jaeckel, Thorsten Lieke

**Affiliations:** 1 Department of Gastroenterology, Hepatology and Endocrinology, Hannover Medical School, Hannover, Germany; 2 Department of General-, Visceral- and Transplantation Surgery, Hannover Medical School, Hannover, Germany; 3 Bernhard Nocht Institute for Tropical Medicine, Parasitology Section, Hamburg, Germany; 4 ReMediES, Department of General-, Visceral- and Transplantation Surgery, Hannover Medical School, Hannover, Germany; University of Nebraska Medical Center, United States of America

## Abstract

**Background:**

T cells are known to participate in the response to tumor cells and react with cytotoxicity and cytokine release. At the same time tumors established versatile mechanisms for silencing the immune responses. The interplay is far from being completely understood. In this study we show contacts between tumor cells and lymphocytes revealing novel characteristics in the interaction of T cells and cancer cells in a way not previously described.

**Methods/ Findings:**

Experiments are based on the usage of a hydrophilic fluorescent dye that occurs free in the cytosol and thus transfer of fluorescent cytosol from one cell to the other can be observed using flow cytometry. Tumor cells from cell lines of different origin or primary hepatocellular carcinoma (HCC) cells were incubated with lymphocytes from human and mice. This exposure provoked a contact dependent uptake of tumor derived cytosol by lymphocytes – even in CD4^+^ T cells and murine B cells – which could not be detected after incubation of lymphocytes with healthy cells. The interaction was a direct one, not requiring the presence of accessory cells, but independent of cytotoxicity and TCR engagement.

Electron microscopy disclosed 100-200nm large gaps in the cell membranes of connected cells which separated viable and revealed astonishing outcome. While the lymphocytes were induced to proliferate in a long term fashion, the tumor cells underwent a temporary break in cell division. The *in vitro* results were confirmed *in vivo* using a murine acute lymphoblastic leukemia (ALL) model. The arrest of tumor proliferation resulted in a significant prolonged survival of challenged mice.

**Conclusions:**

The reported cell-cell contacts reveal new characteristics i.e. the enabling of cytosol flow between the cells including biological active proteins that influence the cell cycle and biological behaviour of the recipient cells. This adds a completely new aspect in tumor induced immunology.

## Introduction

Cancer is like hide-and-seek between tumor cells and the immune response. The immune system when challenged by cancer, however, is faced with the problem that MHC self-expressing cells need to be controlled in their malignancy. Nevertheless, the switch of normal cells into tumor cells is accompanied by the expression of tumor specific peptides able to activate T cells (reviewed by [1]). Most of those peptides descended from proteins not exclusively produced by tumor cells but modified in their structure. The T cell response keeps the tumor in a steady or dormant state [2,3]. It has been an accepted hypothesis that most of the anti-tumor responses are mediated by CD8^+^ T cells and CD4^+^ T cells are restricted either to help CD8^+^ T cells for effective cytotoxicity [4,5] or prime dendritic cells (DC) to enhance the response of CD8^+^ T cells [6,7]. 

In contrast to this dogma recent reports revealed participation of CD4^+^ T cells as powerful effector cells with capacity for direct action against tumor cells leading to regression of the tumor [8–10]. It has been shown that transfer of tumor-antigen specific CD4^+^ T cells in challenged but immune-deficient mice can cause complete tumor regression without the need of CD8^+^ T cell, NK cell or B cell assistance [10]. However, the presumption for all described powerful T cell responses is either a transgenic specificity of the T cell receptor (TCR) against known tumor-antigens or isolation and expansion of tumor-infiltrating lymphocytes (TIL). 

Thus, activation of the immune response can be observed but in the course of tumor growth an editing of the immune response occurs. This includes equilibration and finally immune escape of tumor cells by induction of resistance [11]. This efficient immune evasion of tumors is due to creation of a microenvironment that attracts suppressive myeloid-derived cells and regulatory T cells. In addition the cytokine and chemokine composition as well as the expression of certain ligands on tumor cells may convert effector cells into regulatory cells or drive them into anergy and apoptosis (reviewed by [11,12]). The knowledge of this back and forth of the immune system and cancer *in vivo* is still full of gaps thus every additional interaction of T cells with tumor cells helps to understand the response and escape mechanisms.

In this study we report of a so far not described interaction between tumor cells and T cells, both CD4^+^ and CD8^+^ T cells. This includes contact formation with different characteristics from the immunological synapse. The formation of the synapse has been extensively investigated and involves several stages including pseudopodia, microtubule formation and co-localization of mitochondria and endoplasmic reticulum [13]. All these attributes are missing in the contacts we observed. Instead the contacts leading to cytosol exchange between lymphocytes and tumor cells *in vitro* and *in vivo* induce a break in proliferation of the tumor cells. In contrast to previous reports these contacts occur independently on the presence of antigen-presenting cells. As a consequence of these interactions, ongoing T cell division is induced in lymphocytes which incorporate tumor derived cytosol. This specific phenomenon is a new aspect of the interplay between the immune system with tumor cells. 

## Results

### Contact formation between tumor cells and T cells induce flow of cytosol unassociated with cytotoxocicity

**Figure 1:**
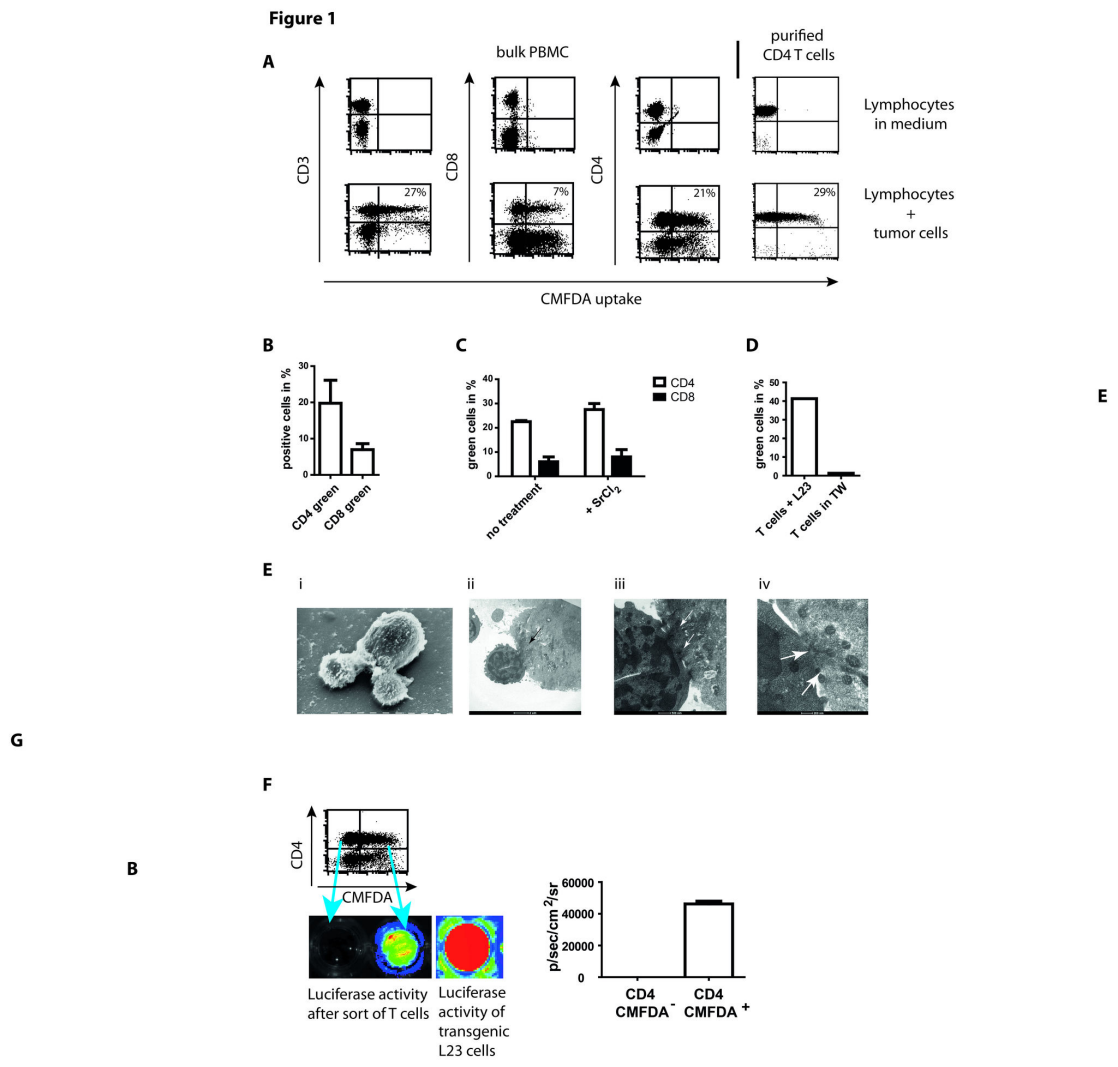
Exchange of cytosol is mediated by contacts between purified CD4^+^ T cells and tumor cells and associated with transfer of enzymes of high molecular weight. (A) 2x10^6^ human PBMCs or purified CD4^+^ T cells were incubated with 2x10^6^ CMFDA-labeled L23 cells for 4 h, subsequently stained for CD3, CD4 and CD8 and analyzed in flow cytometry, respectively. The exposure of lymphocytes to L23 cells led to an inclusion of fluorescence in numerous T cells. (B) Summary of four independent experiments performed as described above. The frequency of green fluorescent T cells is related to bulk population of PBMCs. (C) PBMCs were pre-incubated with 5mM strontium chloride to induce degranulation before the exposure to L23 cells. Adpation of fluorescence was assessed in CD4^+^ and CD8^+^ T cells. (D) 2x10^6^ purified T cells and 2x10^6^ L23 were incubated for 4 h together or separated by transwells (TW), the labeled L23 cells at the bottom of a 96well round-bottom plate and T cells placed in the transwells. (E) Purified CD4^+^ T cells were incubated with L23 cells for 4 h, fixed and prepared for electron microscopy (EM). Scanning (i) and transmission EM (ii-iv) revealed intense contacts. In transmission EM flow of cytosol was visualized by the electron dense cytosol of lymphocytes which distributed from the site of contacts in the tumor cell (arrows in (ii) and (iii)). In the highest magnification gaps in the cell membranes could be detected (arrows in iv). The gaps dehisced length up to 200nm allowing free flow of cytosol and soluble components but not of cellular structures e.g. mitochondria. However, the aggregation of mitochondria in the area of contact formation might be an indication of energy consuming processes. (F) CD4^+^ T cells were sorted after incubation with CMFDA-labeled luciferase-transgeneic L23 cells in green-fluorescent and non-fluorescent T cells. 5x10^4^ separated T cells were subsequently incubated in medium supplemented with 100µg/ml luciferin in 96well round-bottom plates. Luminescence was assessed after 30 min incubation using IVIS analysis. 5x10^4^ transgeneic L23 cells served as control of luminescence intensity. Results are shown as representative pictures and scatter-grams or summarizing 4 independent experiments as mean ± SEM.

A brief note: The experiments presented in Figure 1 show the interaction of human PBMCs with the porcine B cell leukemia line L23. Initially we asked if cells of this porcine cell line are targets for human NK and cytotoxic T cells and intended to analyze this in flow cytometry as we have performed previously using parasites as targets for NK cells [14,15]. As a coincidence we found the adaption of fluorescence by T cells after exposure to L23 cells. It further turned out that this effect is not a xenogeneic specific phenomenon but can be observed with human and murine lymphocytes and several tumors of different origin. However, this model revealed the most significant results and thus is best suited to reflect this novel interaction between T cells and tumor cells. We are aware of the artificial system since human PBMCs and porcine tumor cells are unlikely to be found together under natural conditions. Thus, we limited the data using porcine cells to Figure 1 and subsequently switched to a mouse model.

For fluorescent labeling of cells we used CellTracker Green (5-chloro-methylfluorescin diacetate [CMFDA]). CMFDA initially is a non-fluorescent molecule able to pass the cell membrane. In viable cells CMFDA is cleaved into the fluorescent, strongly hydrophilic form building a bulky water sheath. This averts its release through the intact cell membrane of viable cells. CMFDA can be used as a marker for viability since in case of cytotoxic lysis, labeled cells become non-fluorescent because of loss of CMFDA-containing cytosol [14]. An analysis in flow cytometry is feasible if effector and target cells differ markedly in size and thus can be distinguished in the forward-sideward-scatter (Figure **S1**). However, instead of loss of fluorescence we observed an uptake of fluorescence by lymphocytes after co-culture of tumor cells with PBMCs indicating a transfer of CMFDA molecules from one cell to the other (Figure **1A**). The adaption of fluorescence was almost exclusively restricted to T cells since only a very small fraction of CD3^-^ cells became fluorescent. Of note, the observed interaction of T cells with tumor cells required no bystander cells as exemplified by exposure of highly purified CD4^+^ T cells to L23 cells. Also this setting induced a pronounced ratio of green-fluorescent cells (Figure **1A**). 

As the dot blot shows, this interaction pertained to a considerable number of T cells: On average 20% of the CD4^+^ and 7% of CD8^+^ T cells among bulk PBMCs became green fluorescent (Figure **1B**). This phenomenon can only be explained by the uptake of cytosol containing the CMFDA molecules from one cell to the other. It is remarkable that NK cells revealed no signs of gathering fluorescent tumor derived cytosol although they exhibit strong cytotoxic activity against the L23 tumor cells (Figure **S2**). However, cytotoxicity appears to be a reasonable explanation for the observed effect. We tested the possibility of cytotoxicity being responsible for exchange of cytosol by treatment of PBMCs with strontium chloride (SrCl_2_), a reagent that induces exocytosis of lytic granules [16]. Thus, treatment with SrCl_2_ leaves cytotoxic cells ineffective. Nevertheless, lymphocytes still revealed an uptake of CMFDA (Figure **1C**). This accounts for both CD4^+^ and CD8^+^ T cells which is an indication that cytotoxicity is not the agent for the exchange of cytosol. Indeed, although only slightly we observed an increase of fluorescent adaption after SrCl_2_ treatment in both populations of T cells. However, contact formation was obligatory for the effect observed. T cells and tumor cells were incubated either in co-culture or separated by transwells. Green fluorescent T cells could only be detected after direct exposure to tumor cells but not in transwell cultures (Figure **1D**). 

### Gaps in the cell membranes of tumor cells and naïve lymphocytes allow the transfer of cytosol including high molecular weight molecules and induce proliferation of T cells

We further focused on the interaction of CD4^+^ T cells with tumor cells by visualization of the contact formation. Scanning electron microscopy (SEM) revealed the intense nature of the cell contacts leading to polarization of the interacting tumor cell-lymphocyte partners (Figure **1E** **i**). Furthermore, transmission electron microscopy (TEM) showed, even at low magnification, a flow of electron dense cytosol from the T cell into the tumor cell which emerged at the highest magnification to be enabled by gaps in the cell membrane of both partners (Figure **1E** **ii-iv**). These gaps extended areas between 100-200nm and thus should enable the transfer of more than just low molecular weight components of the cytosol but also molecules with enzymatic function of high molecular weight. In consequence the passage of the 30kDa protein luciferase was assessed by co-culture of PBMCs with retroviral transduced luciferase expressing L23 cells. Transduced L23 cells were labeled with CMFDA and T cells were separated after exposure to the tumor cells distinguishing the green-fluorescent from the non-fluorescent CD4^+^ T cells. Purified T cells were subsequently incubated with luciferin to assess enzymatic activity. Indeed, T cells with uptake of CMFDA revealed luminescence whereas the non-fluorescent T cells were negative for luciferase activity (Figure **1F**). This clearly showed a transfer of cytosol associated with an exchange of molecules that sustained biological activity in the recipient cell. Thus, it is of interest to follow the consequences of contacts for the partners if cytosolic components maintain their biological function. Basically we did that in the mouse model *in vitro* and *in vivo* but found supporting data also with human lymphocytes and porcine tumor cells.

We separated fluorescent and non-fluorescent CD4^+^ T cells after exposure to CMFDA-labeled tumor cells and isolated cells were cultured without any further stimulation in medium for 5 days. We found pronounced proliferation of those T cells with incorporation of tumor derived cytosol (Figure **S3**). T cell expansion can be induced by T cell receptor dependent and independent pathways. It has been reported that T cells internalize the TCR after exposure to anti CD3 monoclonal antibody (mAb) clone OKT3 under certain conditions [17]. Thus, we applied the protocol in our experiments and verified the disappearance of TCR from the cell surface of T cells. Nevertheless, the uptake of fluorescence was not abrogated by the treatment. Furthermore, blocking of porcine MHC II molecules with mAb MSA-3 left the outcome of cytosol transfer between lymphocytes and tumor cells virtually unaffected. Last but not least, for uptake of cytosol the lymphocytes did not have to be pre-activated. Naïve T cells revealed the capacity to undergo the contacts with tumor cells at least to the same degree as IL-2 activated or x-rayed L23 pre-exposed T cells (**all data shown **in Figure **S3**). 

### Exchange of cytosol is not restricted to a certain species and type of tumor cells

Switching to the mouse model, we used a highly aggressive tumor cell line BM185, isolated from BALB/c mice suffering from ALL [18], to validate the universality of the observed contacts between T cells and tumor cells. The expansion in the mouse model offers additionally the possibility of *in vivo* verification. 

**Figure 2:**
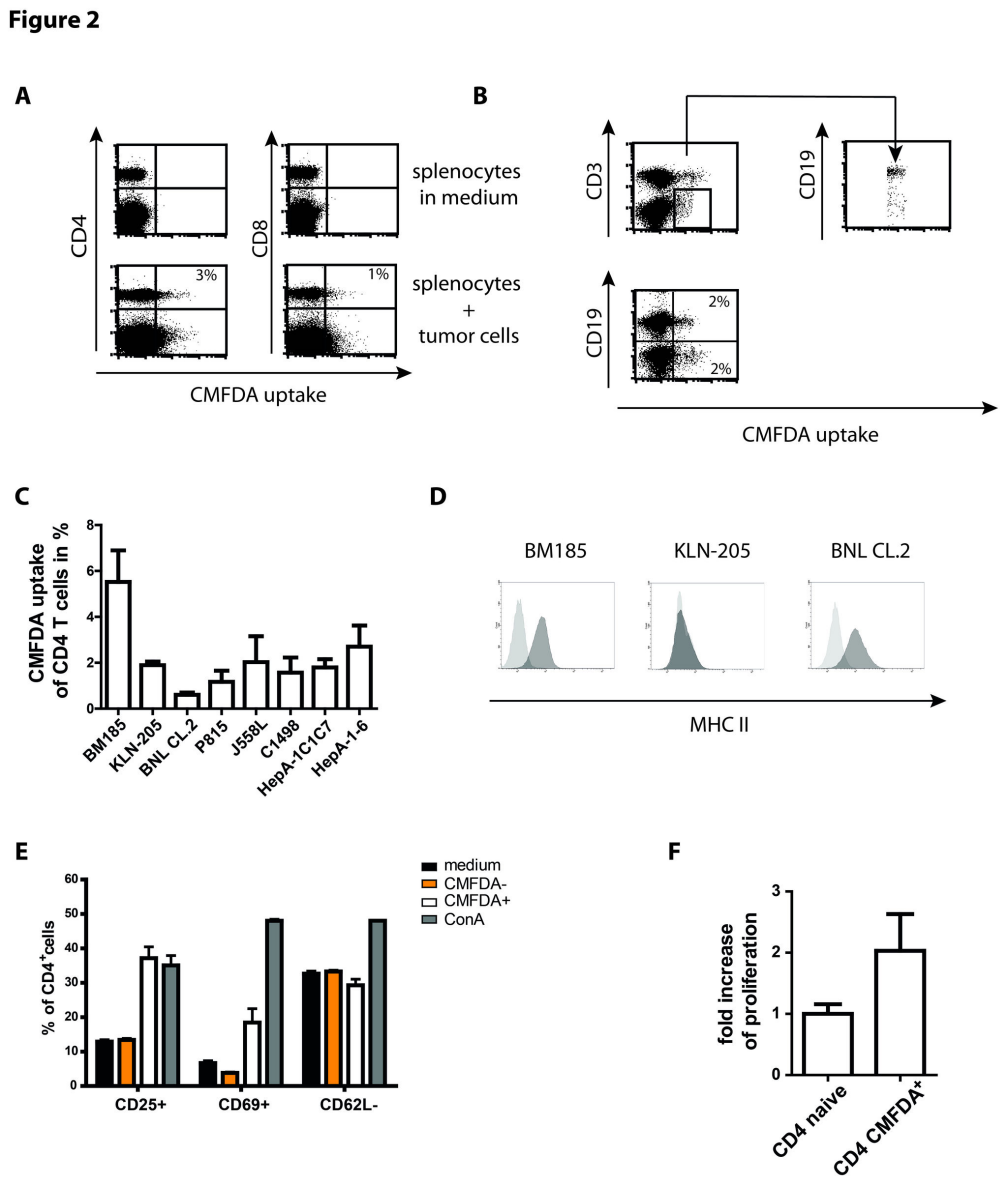
Murine T cells exchange cytosol with tumor cells and reveal signs of activation. (A) 2x10^6^ splenocytes incubated with 2x10^6^ CMFDA-labeled BM185 cells for 4 h and analyzed for fluorescence uptake by T cells. Likewise to the human PBMCs the mouse model revealed green-fluorescent CD4^+^ and CD8^+^ T cells. In addition, a well detectable green-fluorescent population extending the T cell population could be observed within the splenocytes. (B) Staining of splenocytes with CD3 and CD19 after exposure to tumor cells identified the non-T cell population capable for cytosol uptake as B cells. (C) Summary of four independent incubation experiments of splenocytes with cells of different cell lines. BNL cells are not tumor derived whereas the other lines are tumor cells. (D) Analysis of MHC II expression of the respective cell lines BM185, KLN-205 and BNL CL.2. The level of expression did not correlate with the outcome of cytosol transfer. (E) In contrast to unaffected CD4^+^ T cells (CMFDA^-^), CD4^+^ T cells revealing an uptake of tumor derived cytosol (CMFDA^+^) showed signs of activation according to increased expression of CD25 and early activation marker CD69 whereas CD62L slightly decreased. Cells were incubated as described above (A) with subsequent staining of CD4 T cells and activation markers. The pattern of activation was different to the stimulation with 2µg/ml ConA. (F) Proliferation analysis of 1x10^4^ purified T cells revealing CMFDA uptake after 4 h exposure of splenocytes to labeled BM185 cells. For control purified CD4^+^ T cells of naive mice were cultured in medium as it was performed with sorted T cells for 4 days and additional 16 h with ^3^H thymidine before szintilization. Results are shown as representative scatter-grams or summarizing at least 3 independent experiments as mean ± SEM.

Electron microscopy and flow cytometry analysis confirmed the contacts and transfer of cytosol between murine T cells and BM185 cells (Figure **2A** and Figure **S4**). Of note, in contrast to human PBMCs, among bulk murine splenocytes a well detectable population of CD3^-^ cells revealed the capability to interact with tumor cells resulting in cytosol uptake. Additional staining of splenocytes with CD19 identified the population as B cells by gating on the CD3^-^ cells which were almost completely CD19^+^ (Figure **2B**), whereas in line with the results using human cells, NK cells were not involved in cytosol transfer (**data not shown**).

In general, murine T cells had a less pronounced tendency to interfere with tumor cells in comparison to human T cells. Even with L23 cells only a mean of 10% CD4^+^ T cells became green fluorescent and this coupled with a very high standard deviation. Nevertheless L23 cells induced the highest uptake of CMFDA (**data not shown**). In addition, other murine cell lines including the KLN-205 lung carcinoma cells, embryonic BNL CL.2 liver cells, mastcytoma cells P815, J558L B cell myeloma cells, C1498 myeloid tumor cells and the liver tumor cell lines HepA-1C1C7 and HepA-1-6 were tested. With the exception of BNL cells, variable but well detectable CMFDA transfer for the different tumor cells was found (Figure **2C**). Interestingly, although permanently dividing, BNL CL.2 cells are not regarded as classical tumor cells [19]. The resistance of BNL CL.2 cells, offered also for the mouse model the opportunity to validate the TCR independency and tumor specificity of the effect. We assessed the levels of MHC II expression on the different cell lines. It turned out that BM185 and BNL CL.2 expressed high level of MHC II while KLN-205 cells were completely negative for MHC II (Figure **2D**). Thus the TCR/MHC interaction seems not to be obligatory for the exchange of cytosol. This is also highlighted by the transfer of cytosol observed between B cells and tumor cells. In addition, we performed experiments using hemagglutinin (HA)-transduced BM185 cells and compared the outcome of cytosol transfer after exposure to wild-type (wt) BALB/c and transgenic 6.5 mice. The 6.5 mouse has a characteristic population of about 20% of CD4 T cells expressing a HA-specific TCR. Both strains revealed a tendency of increased frequency of cells showing exchange of cytosol after exposure to BM185-HA in comparison to BM185 cells. However, splenocytes derived from 6.5 mice did not show increase frequency of cells showing exchange of cytosol with the HA-transgenic BM185 cells (Figure **S5**). 

However, after exposure to tumor cells T cells showed early signs of activation. The incubation of splenocytes with BM185 resulted in up-regulation of the activation markers CD25 and CD69 and marginal alteration of expression of CD62L in T cells revealing an uptake of tumor derived cytosol whereas T cells without adaption of fluorescence showed expression levels of activation markers comparable to medium incubated lymphocytes (Figure **2E**). The detected levels of CD25 on medium incubated and CMFDA^-^ T cells was due to constitutive CD25 expression on regulatory T cells thus levels increased over this background were signs of activation. The assessed activation of T cells is combined with proliferation of the lymphocytes just as could be observed with human T cells interacting with L23 cells (Figure **2F**). 

### Detectable transfer of cytosol in vivo

**Figure 3:**
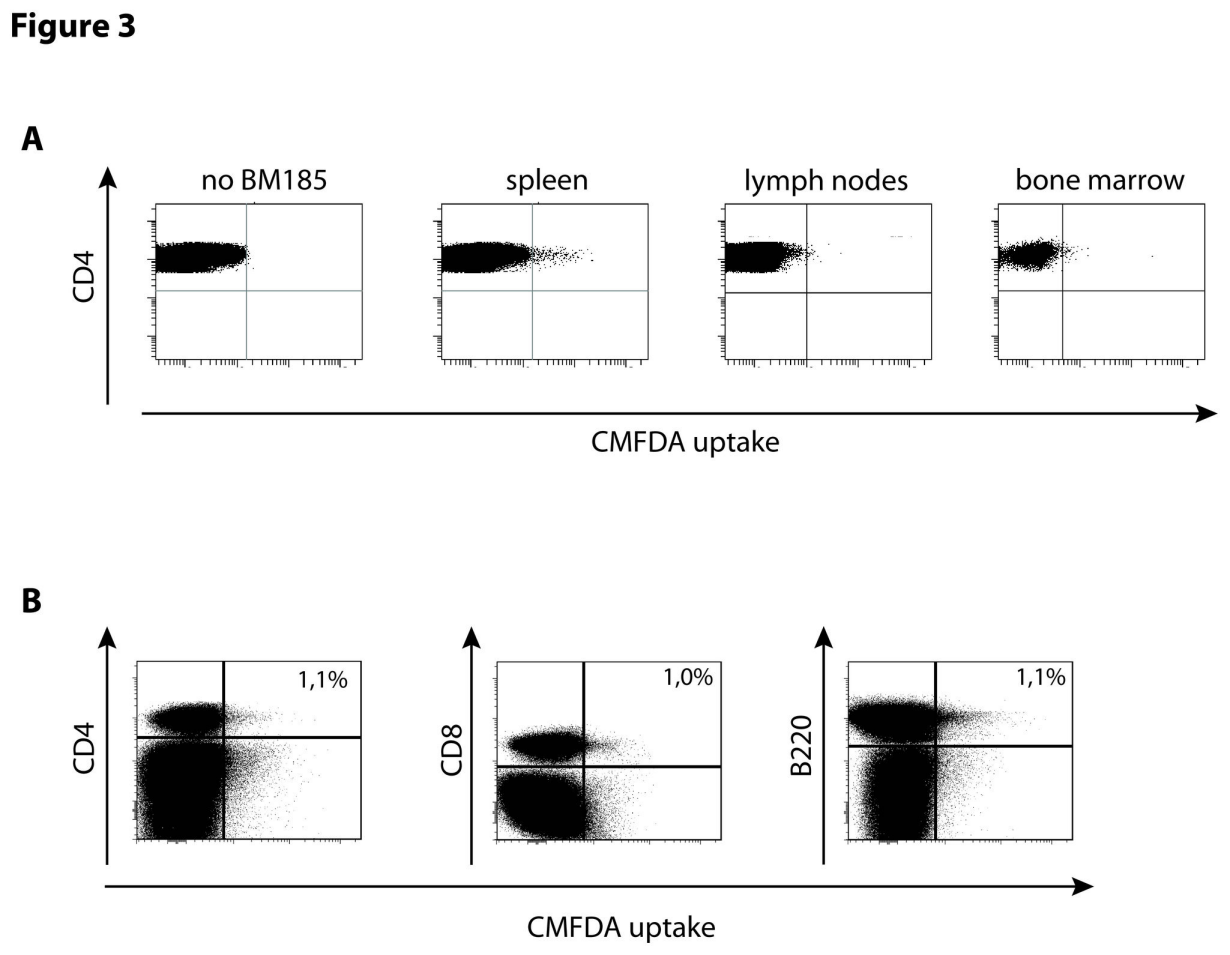
The uptake of cytosol by T cells occurs *in vivo*. (A) 1x10^7^ CMFDA-labeled BM185 were injected intravenously. 6 h later mice were sacrificed and spleen, lymph nodes and bone marrow was homogenized. 5x10^7^ cells were stained for CD4 and counted in flow cytometry and the proportion of green fluorescent lymphocytes was assessed. (B) Assessment of the phenotype of green-fluorescent splenocytes 6 h after i.v. application of 1x10^7^ CMFDA-labeled BM185. In addition to CD4^+^ and CD8^+^ T cells, also *in* *vivo* B cells (B220^+^ cells) revealed CMFDA uptake. Results are shown as representative scatter-grams of 3 independent experiments.

The verification of cytosol transfer *in vivo* is of high interest to exclude the possibility that the cytosol exchange is only due to an *in vitro* artifact. Therefore, CMFDA-labeled BM185 cells were injected intravenously (i.v.) into BALB/c mice and the mice were sacrificed 6 h thereafter. Subsequently spleen, lymph nodes and bone marrow were checked for BM185 cells but only in the spleen could numerous tumor cells be detected (**data not shown**) which was combined with the appearance of green-fluorescent CD4^+^ T cells (Figure **3A**). In line with the *in vitro* results, also *in vivo* lymphocytes with the affinity of cytosol uptake could be found in the populations of CD8^+^ T cells and B cells (Figure **3B**). 

### Tumor cells stop proliferating after uptake of T cell derived cytosol which leads to prolonged survival of mice

**Figure 4:**
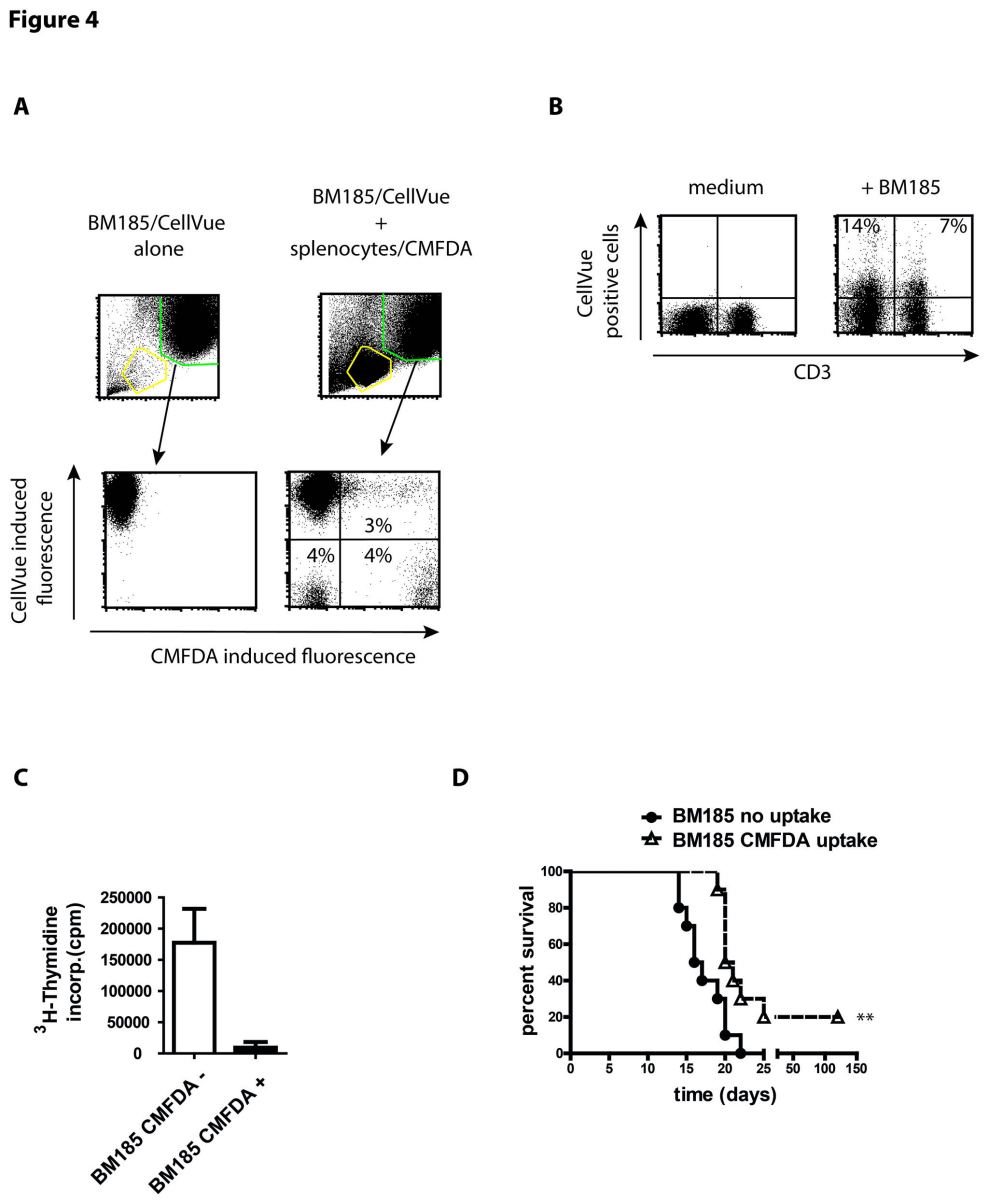
Uptake of cytosol by tumor cells from lymphocytes causes arrest of cell cycle and prolonged mouse survival. (A) BM185 cell were labeled with CellVue to distinguish the population of tumor cells from CMFDA-labeled lymphocytes. Tumor cells and splenocytes were incubated in equal number for 4 h and uptake of CMFDA was assessed by flow cytometry. Further on, BM185 were separated by cell sorting in non-fluorescent and fluorescent cells. (B) Assessment of transfer of membrane components from CellVue labeled BM185 to lymphocytes after exposure of cells in a ration 1:1 for 4 h. CellVue incorporates into lipid regions of cell membranes. (C) ^3^H thymidine incorporation of BM185 cells exposed to CMFDA-labeled splenocytes from BALB/c and subsequently separated by FACS cell sorting in tumor cells which exchanged cytosol with mouse lymphocytes (CMFDA^+^) or those that remained unaffected (CMFDA^-^). Cells were cultured for 3 days in 96 well plates and 16 h after addition of radioactive thymidine. (D) Survival curve of BALB/c mice after i.v. injection of 1x10^4^ BM185 cells (5 mice per group/experiment, the survival curve represents two independent experiments, thus n = 10) with or without uptake of CMFDA after exposure to CMFDA-labeled murine splenocytes. The course of the BM185 elicited leukemia is highly reproducible. The survival of the BM185 cells without the uptake of lymphocyte derived cytosol corresponds completely to this known decline of viablitiy. In contrast, mice injected with BM185 incorporated splenocytes derived cytosol survived significantly longer (p ≤ 0.0042). Results are shown as representative scatter-grams or summarizing 2-4 independent experiments as mean ± SEM.

The flow of cytosol is not a one way road but can be observed in both directions. Thus, not only T cells became green-fluorescent after exposure to CMFDA-labeled tumor cells but also a proportion of tumor cells became green-fluorescent after incubation with CMFDA-labeled lymphocytes (Figure **4A**). To follow the transfer of lymphocyte derived cytosol BM185 cells were labeled with CellVue Maroon, a dye which is incorporated in lipid regions of membranes with the highlighted remark of the manufacturer of minimal unspecific transfer between cells. By exposing the CellVue-labeled tumor cells to lymphocytes we were able to detect a positive fraction with CellVue induced fluorescence on CD4^+^ T cells indicating a transfer of parts of cell membrane of the tumor cells to the surface of lymphocytes (Figure **4B**). This is convincing validation of the impression of the fusion of cell membranes as demonstrated by TEM (Figure 2 and Figure S4). 

We separated BM185 cells into fluorescent and non-fluorescent after exposure to murine splenocytes. The isolated tumor cells were cultured for 4 days with subsequent assessment of ^3^H-thymidine incorporation. Surprisingly, a drastic decrease of proliferation of BM185 cells after absorption of lymphocyte derived cytosol could be measured (Figure **4C**). This was only a temporary discontinuation of cell cycle. 7 days of culture revealed well detectable proliferation even of the tumor cells that underwent transfer of cytosol (**data not shown**). 

The break of tumor cell division might have possible consequences for the survival of BALB/c mice. Of note, BM185 cells are highly malignant causing an aggressive course of ALL. Even a low dose of 10^4^ cells leads to death within 20 days. BM185 cells that appeared non-fluorescent after exposure to splenocytes revealed a highly reproducible course of mortality. In contrast, mice injected with BM185 that included cytosol from splenocytes survived significantly (p ≤ 0.0042) longer and 20% of mice (two out of ten) even survived the challenge with tumor cells (Figure **4D**). 

### T cells exchange cytosol specifically with tumor cells but not with healthy cells

The issue remains: Is this an exclusive interaction of T cells with tumor cells? And if so: Is the effect restricted to tumor cell lines or do primary tumor cells also induce this phenomenon? The latter question is of interest since cytosol transfer with primary tumor cells would confirm clinical importance special when considering that the immune system is activated by the cell contacts with tumor cells and the tumor reveals a temporary arrest in cell division.

**Figure 5:**
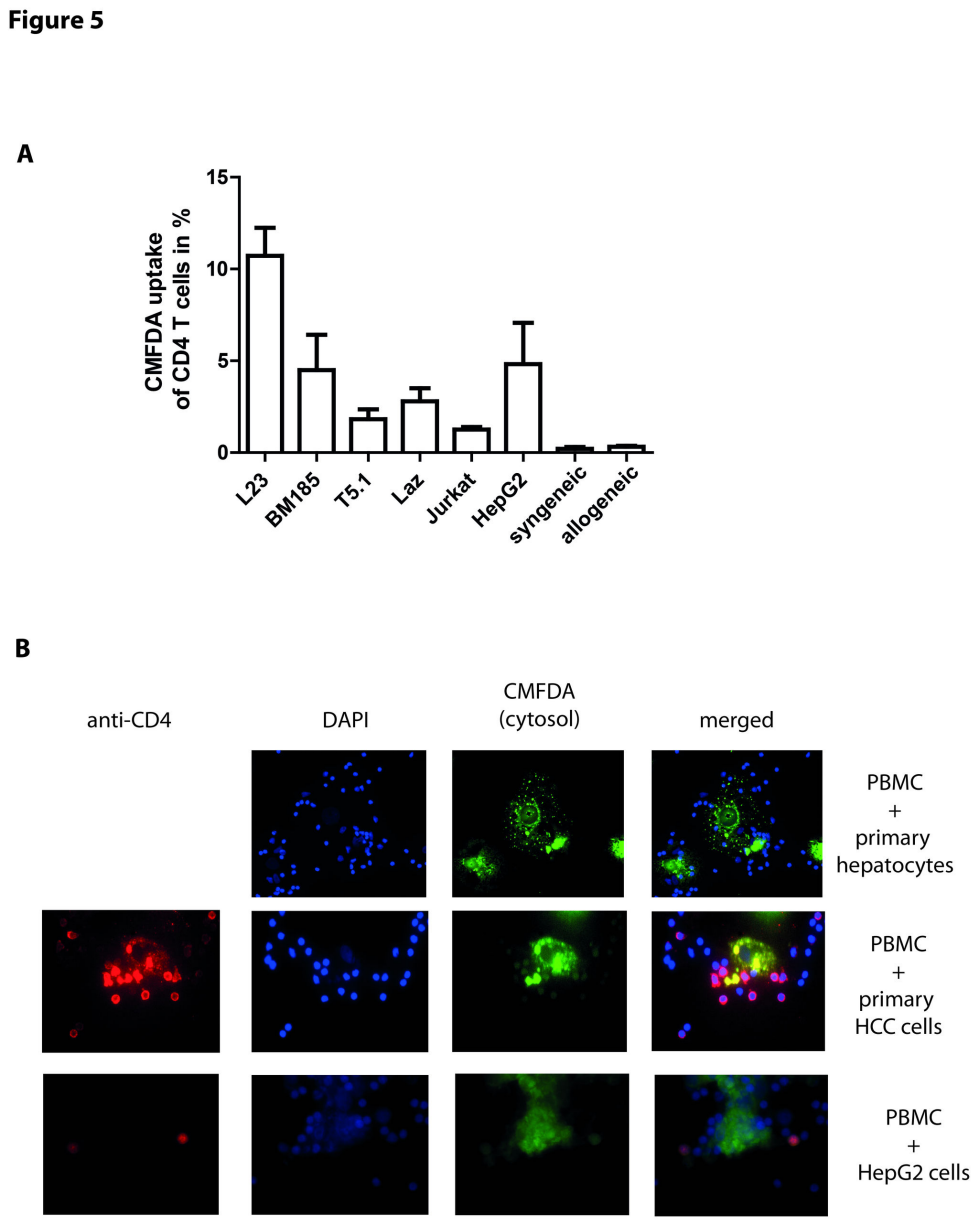
The uptake of cytosol occurs between T cells and tumor cells but not with healthy cells. (A) Exposure of PBMCs to different CMFDA-labeled tumor cell lines including the xenogeneic porcine L23 cells and murine BM185 cells as well as the human B cell lymphoma T5.1 and Laz, Jurkat T cell line and the hepatocarcinoma cells HepG2. With all tested tumor cell lines a transfer of cytosol was assessed. In contrast, human PBMCs exposed to healthy CMFDA-labeled PBMCs from the same blood donor (syngeneic) or different blood donor (allogeneic) showed no adaption of fluorescence. (B) Primary hepatocytes derived from healthy tissue or HCC and HepG2 cells were cultured for 3 days in Chamber Slides to obtain a loose monolayer of cells. Hepatocytes were labeled with CMFDA and incubated with 5x10^5^ PBMCs for 4 h. Cells were fixed and air-dried with subsequent staining for CD4 (red; only HCC and HepG2) and the nucleus (blue). Microscopy revealed an uptake of green fluorescence by lymphocytes only if exposed to tumor cells. Additional staining of CD4 confirmed that some of the participating cells are CD4^+^ T cells. Results are shown as representative pictures and scatter-grams or summarizing 3 independent experiments as mean ± SEM (except for (B) which summarizes at least 6 experiments).

As performed with murine splenocytes, experiments were repeated by exposing different tumor cell lines to human PBMCs. We chose BM185 cells as an additional xenogeneic tumor line, human B cell lymphoma cells and the liver tumor cell line HepG2. In comparison to L23 cells the effect was less pronounced in all tested cell lines. However, a clear uptake of cytosol by CD4^+^ T cells after co-culture with all tumor cell lines could be observed (Figure **5A**). Interestingly, BM185 cells also induced within human PBMCs an adaption of fluorescence in 5% of CD4^+^ T cells from bulk PBMC just as with murine syngeneic splenocytes. Thus, the distinct exchange of cytosol with L23 cells cannot be solely explained by the xenogeneic setting. 

Thus, tumor cells induced the exchange of cytosol. To control if this effect was specific for tumor cells human PBMCs were incubated with CMFDA-labeled allogeneic PBMCs or autologous PBMCs derived from healthy blood donors. We did not find uptake of CMFDA comparable when tumor cells were used (Figure **5A**). Of note, splenocytes from Lewis rats bred under specified-pathogen free (SPF) conditions did not provoke adaption of fluorescence in human PBMCs, too (**data not shown**).

To exclude the possibility that this effect was caused by tumor cell lines which are often induced and stabilized by viruses (as for L23 cells, [20]) primary tumor cells derived from human HCC were cultured with allogeneic PBMCs. Since we excluded the possibility of cytosol transfer between allogeneic donor cells, the observed effects are interpreted to be related to the tumor cells. Using microscopic techniques a vigorous uptake of CMFDA after exposure of PBMCs to HCC cells was visible. As control, healthy primary human hepatocytes were incubated with PBMCs which did not induce a transfer of fluorescence (Figure **5B**). Furthermore, the results gained with HepG2 cells in flow cytometry were confirmed in microscopic analysis. These results indicate a specific phenomenon of T cells with tumor cells but not with healthy cells, independent of the origin of the tumor.

## Discussion

**Figure 6:**
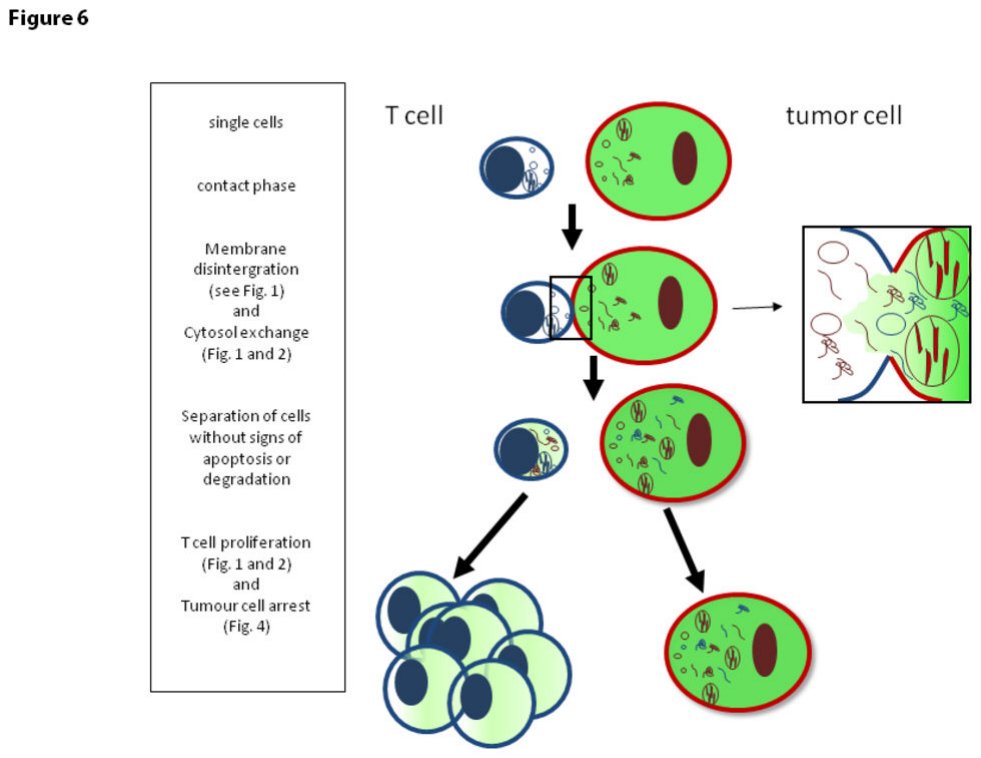
Scheme of the probable course of contact formation and cytosol exchange. The plot shows the process of connection of cells including the membrane disintegration and flow of cytosol. Based on our data, we presume that beside the flow of liquid cytoplasm, proteins, mRNA and small vesicles of the connected cell partners are exchanged. Mitochondria, however, remain in the respective host cell but can be observed in high density in the site of contact formation, indicating energy consuming processes (see Figure 1E; iv).

In this study, we report of a so far undescribed interaction between tumor cells and CD4^+^ T cells. After contact formation the intactness of cell membranes is disrupted and stunningly large gaps allow the stream of cytosol from one cell to the other in both directions. Accompanied with this exchange of fluid, heavy weight molecules with high biological activity are transferred with consequences for both interacting partners. While tumor cells reveal a temporary break in proliferation, T cells start to expand – and this without an involvement of TCR-antigen recognition (summarized in Figure **6**).

A few years ago, Stinchcombe et al. reported of a similar phenomenon between CTL and target cells. In addition to the cytolytic processes that depend on the formation of the immunological synapse they found contacts independent of antigen recognition by the TCR leading to membrane bridges between the partners [21]. These structures had an inner diameter of up to 100 nm which should definitely be large enough to allow a flow of cytosol, however the authors did not report any exchange of cytosol. This might be due to the fact that CFSE was used to assess a transfer of cytosolic components. CFSE probably is not the appropriate reagent since it binds covalently to proteins that often interact with intracellular components e.g. membranes or organelles like the Golgi or endoplasmic reticulum.

Another type of connection between (immune) cells is tunnelling nanotubes [22–24]. They have been described to allow a slow passive transfer of cytosol which, however, is massively impeded by the length of tunnelling nanotubes and the actin filaments in the lumen [24]. In contrast, a recent report demonstrated the transfer of cytosolic components between T cells via nanotubes [25], nevertheless the structure connecting the partner cells span a distance of almost a cell diameter.

The gap-formation described in the present study, however, did not resemble on membrane bridges or tubes. Rather, they showed the phenotype of holes, tight as gap junctions, besides which, we did not find the connexin ring forming the junction. Therefore, we propose a novel type of connection between T cells and target cells. TEM analysis suggests a temporary fusion of cell membranes which reverts in normal cell membranes leaving the respective partner viable.

The outcome of the transfer of cytosol is astonishing: The T cells start to proliferate and the tumor cells undergo a temporary cell cycle arrest. Both events imply an interposition in transcription of genes affecting the division of cells. A possible explanation is the transfer of components with the ability to affect the activity of transcription factors. Transcription factor activity depends on a number of molecules involved in signal transduction e.g. extracellular regulated kinases which are located in the cytosol. This raises the question if such a short termed exchange of molecules can induce a long lasting effect with such drastic changes within the cell cycle. 

The activation of T cells by tumor cells is – so far reported – TCR/MHC mediated and after a short period of activity against the tumor cells leads to tolerance [26]. This is supported by several studies reporting that local suppression of the immune response is restricted to primary tumors [27–29]. Our results indicate a different pathway of activation. However, it remains unclear which molecules on the surface of both tumor cells and lymphocytes mediate the contacts between the cells. The percentages of T cells that obviously interfered with tumor cells suggest an ubiquitary expression of interacting molecules on both, tumor cells and lymphocytes. It is important to note that these contacts leading to such dramatic consequences occur only in the presence of tumor cells. Tumor cells reveal several modifications in molecules, especially in the glycosylation of proteins [30]. It is reasonable to assume that this switch in molecule characteristics facilitates contact formation. 

In a series of experiments we assembled evidence that contact formation and induction of membrane connection is not dependent on TCR/MHC recognition. Using a transgenic antigen-specific mouse model, we showed that the effect remained almost unaffected by TCR down-regulation, MHC II blockage or MHC II expression in general. 

We tested the involvement of classical molecules expressed on T cells enabling cell-cell contacts of T cells with targets as reported for selectins [31] and integrins [32] as well as the involvement of CD2 [33] but none of these molecules were inevitable for the exchange of cytosol (data not shown). The data suggest an expression of a molecule of unspecific nature since in the mouse model, the molecule expression seems to be common for both T and B cells. 

The involvement of CD4^+^ T cells was unexpected. In general, the participation of CD4^+^ T cells in immune responses against cancer was assumed to be restricted to a helping manner for the generation of powerful CTL activity [4,5]. However, CD4^+^ T cells have been recently reported to act directly against tumor cells by elimination of the targets [8–10]. Of note, these T cells have to be pre-exposed to leukaemia cells and thus require the activation through APC [9] or express specific TCR for prominent oncogenes by transgeneic modification [8,10]. 

In contrast, our study opens the possibility that interaction of T cells with tumor cells occurs without any accessory cells and does not depend on prior activation. We explicitly excluded the need for this requirement by activating PBMCs in various ways. The comparison of percentages of naïve or pre-activated T cells with tumor derived cytosol incorporation revealed no difference. 

Assessment using the mouse model confirmed the exchange of cytosol *in vivo*. In addition, the *in vivo* experiments showed effects on the course of tumor progression after contacts with T cells. Mice exposed to tumor cells with uptake of T cell derived cytosol disclosed a prolonged survival which is associated with decelerated expansion of absolute numbers of tumor cells (data not shown). The data revealed significance because of an extremely predictable course of tumor progression as it has been reported earlier [34] and reproduced in our own lab. 

Noteworthy, with the BM185 cells we used a highly aggressive tumor cell line with a progressive decline in vitality of challenged mice. The break of tumor cell division after the interaction with lymphocytes is – although only short-term – sufficient to control the tumor at least at the beginning, in the period of tumor establishment. That this effect is only obvious after cell sorting and is concealed by injection of bulk tumor cells is most likely due to the low frequency of tumor cells interacting with lymphocytes thus are of no consequences in terms of normally observed tumor progression. Thus, the implication of the exchange of cytosol for the interplay of immune system and tumor *in vivo* needs to be further evaluated. 

However, we were able to demonstrate that the exchange of cytosol is not an artificial mechanism with immortalized tumor lines. Since tumor cell lines are often stabilized by viruses for permanent growth *in vitro* it could be argued that virus derived molecules are expressed on the surface of tumor cells [35,36] mediating the contacts and the described effect is not linked to cancer at all. We showed that this possibility is not consistent with our data because primary tumor cells derived from patients suffering from HCC exchanged cytosol with T cells in the same manner. Thus, this newly described interaction may indeed be of clinical importance. 

## Materials and Methods

### Ethics Statement

Animal care and experiments were conducted in accordance with institutional and national guidelines. All animal experiments were performed according to protocols approved by the animal welfare commission of the Hannover Medical School and the local Ethics Animal Review Board (Niedersächsisches Landesamt für Verbraucherschutz und Lebensmittelsicherheit / LAVES, Oldenburg, Germany). The grant number covering the experiments is 10/0071.

Written informed consent was obtained from human blood donors and patients suffering from HCC. The signed consent forms are documented and archived in the Department of General-, Visceral- and Transplantation Surgery, Hannover Medical School, Hannover, Germany. Ethical approval was received from the local ethical board of the Hannover Medical School to perform these studies.

### Animals

4-6 weeks old Balb/c mice were purchased from Charles River. Transgeneic mice expressing HA-specific T cell receptors of the strain C;Tg(Tcra,Tcrb)1Vbo (from here designated as 6.5) as well as Lewis rats were bred in the animal facility of the Hannover Medical School. 

### Animal experiments

For analysis of survival 5 mice per group were injected intravenously (i.v.) with a total of 1x10^4^ leukaemia cells and the well-being of the mice was checked daily. For assessment of cytosol transfer *in vivo* mice received 1x10^7^ leukaemia cells i.v. and were sacrificed after 6 h. 

### Primary cells and cell lines

Human peripheral blood mononuclear cells (PBMCs) were isolated from donor derived blood-filters containing lymphocytes and granulocytes received from the Department of Transfusion Medicine, Hannover Medical School, using Ficoll gradient centrifugation. Cells were washed and incubated over-night in culture flasks to reduce the percentage of adherent cells.

Primary human hepatocytes were isolated by 2-step-collagenase-perfusion technique from healthy liver specimen as previously described [37]. Primary carcinoma cells were obtained from small tissue samples after surgical removal of HCC as described in brief: Tissue was cut in small cubes and digested with Collagenase IV for 2 h at 37°C. Subsequent after cautious shaking the supernatant was transferred in a new tube and cells were centrifuged at 200 g. Cells were washed and resuspended in DMEM medium + L-glutamine (584mg/l) (Lonza, Cologne, Germany) supplemented with 10% heat-inactivated fetal calf serum (FCS), 0.01 mM non-essential amino acids, 0.05 mM 2-mercaptoethanol, 1 mM sodium pyruvate and antibiotics.

In addition we used tumor cell lines of different origin and species for the experiments. Cells were either incubated in DMEM or RPMI medium supplemented with ingredients as described above and maintained by permanent passages *in vitro*. The following cells lines were included: human cell lines HepG2 liver cells, LAZ 221 and T5-1 B cell lymphoma cells and Jurkat T cells as well as murine cell line adherent KLN-205 lung carcinoma cells, BM185 B cell lymphoma cells, mastocytoma cells P815, J558L B cell myeloma cells, C1498 myeloid tumor cells, the liver cell lines HepA-1C1C7 and HepA-1-6 and furthermore as not-tumor cells embryonic liver cell line BNL CL.2. 

### Fluorescent labeling of cells

a) CMFDA

#### Non-adherent cells

5x10^7^-1x10^8^ human PBMCs, murine splenocytes, L23 and BM185 cells respectively were washed in serum-free medium followed by labeling with 4 µM CellTracker Green (5-chloro-methylfluorescin diacetate [CMFDA]; Molecular Probes, Eugene, Oregon, USA) for 20 min at 37°C. Cells were subsequently washed in warm RPMI medium containing 10% human serum and were further incubated for 30 min at 37°C. After a single final wash labeled cells were incubated with non-labeled cells. This was performed with labeled tumor cells with non-labeled lymphocytes and vice versa.

#### Adherent cells

Culture medium of adherent cells was discarded and cells were washed twice with FCS-free medium. Afterwards, cells were covered with FCS-free medium supplemented with 4µM CMFDA and incubated for 20 min at 37°C. Cells were washed three times warm FCS-containing medium followed by 5 min incubation time and then exposed to unlabeled lymphocytes for 4h.

b) Cell-Vue Maroon

5x10^7^ BM185 cells were washed twice in PBS. After centrifugation cell pellet was resuspended in Diluent C and supplemented with 1x10^-6^ M Cell-Vue Maroon (Polyscience, Eppelheim, Germany). The suspension was incubated for 4 min at RT with subsequent addition of PBS containing 1% bovine serum albumin for another 2 min. Cell suspension was transferred to a new tube and washed three times with FCS-containing RPMI medium.

### Antibody staining/blockage

OKT3/TCR staining: PBMCs were stained with biotinylated mAb anti TCR (clone IP26; BioLegend, Uithoorn, The Netherlands) and consequently incubated with mAb OKT3 as it has been described earlier [17]. In brief, PBMCs were incubated 15 min on ice with or without 0,3µg/ml OKT3 and subsequently transferred to 37°C for 1 h. Afterwards, cells were washed and control staining of TCR internalization was performed in the absence or presence of OKT3 by adding PE-conjugated streptavidin. OKT3 treated PBMCs were exposed to equal cell numbers of L23 cells for 4h.

MHC II staining was performed for murine cells with mAb clone P7/7 and for porcine cells with clone MSA-3. Porcine antibody was used for blocking MHC-TCR interactions in a concentration of 10µg/ml.

Activation of murine T cells was assessed by mAb against CD25, CD69 and CD62L (all purchased from BD Bioscience, Heidelberg, Germany).

### Scanning and transmission electron microscopy

2x10^6^ highly purified human and murine CD4^+^ T cells (purity over 98%) were incubated with 2x10^6^ tumor cells for 4h at 37°C. Cells were subsequently fixed with 2% glutaraldehyde in 100mM sodium cacodylate buffer, For scanning electron microscopy (SEM), cells were applied to poly(L-lysine)-coated cover slides (Cellocate; Eppendorf) by sedimentation for 2 h. The samples were fixed again with 1% osmium oxide in 100mM sodium cacodylate buffer for 30 min at 4°C. After repeated washing, cells were dehydrated with increasing ethanol concentrations and finally critical-point dried. Samples were sputtered with gold particles and analyzed in a scanning electron microscope (PSEM 500; Philips, Hamburg, Germany). For transmission electron microscopy (TEM), the cells were treated as described for SEM, dehydrated with graded ethanol solutions but instead of critical-point dried samples were treated with propylene oxide. The cells were embedded using the AGAR-100 kit (Plano, Wetzlar, Germany), 70-nm ultrathin sections were cut (Ultra Cut E; Reichert/Leica, NuBlock, Germany) and counter-stained with uranyl acetate and lead citrate. Sections were examined with a Philips CM 10 transmission electron microscope at an acceleration voltage of 80 kV.

### Production of viral particles

Lentiviral particles were generated by transient co-transfection of 293T packaging cells with transfer vector encoding for GFP and luciferase together with packaging plasmid pMDL-gp-rre, rev expressing plasmid pRSV-rev and the vsv-g encoding plasmid as previously described [38]. Viral titers were determined on A549 cells, yielding vector titers up to 10^7^ transducing units.

### Generation of transgenic tumor cells by lentiviral transduction

L23 and BM185 cells were transduced with lentiviral particles as previously described [38]. In brief, cells were spin infected with generated lentiviral cell-free particles at a multiplicity of infection of five. Transduced cells were expanded for five days followed by FACS sorting according to their GFP expression with a sorting purity of 100%.

### Incubation of lymphocytes with tumor cells for assessment in flow cytometry and sorting

To evaluate the outcome of exposure of lymphocytes to tumor cells, 2x10^6^ bulk PBMCs and splenocytes or isolated human and murine CD4^+^ T cells either fluorescence labeled or untreated were exposed to 2x10^6^ fluorescent or untreated tumor cells of different origin for 4h, respectively. The cells were subsequently washed after incubation time, stained with the respective antibodies for 30 min on ice and either analyzed directly by flow cytometry using a FACS Calibur (BD Bioscience, Heidelberg, Germany) or sorted using a FACS Aria (BD Bioscience). Purification was always checked by reanalysing the sorting results. Following sorting the cells, lymphocytes and tumor cells were incubated for another 3-7 days in 96 well plates to assess proliferative activity *in vitro* or alternatively were injected intravenously in mice. Proliferation was analyzed by measurement of scintillation following [^3^H]thymidine incorporation using a β-counter (LKB Wallac, Turku, Finland).

### Incubation of lymphocytes with tumor cells for assessment in fluorescence microscopy

CMFDA labeled tumor cells cultured in Chamber Slides (Nunc, Rochester, NY, USA) were exposed to 5x10^5^ human lymphocytes for 4h. The ratio of cells co-cultured was difficult to evaluate since the number of adherent cells could hardly be determined. However, titration of cell numbers revealed the appropriate number of cells used for analysis. After incubation cultures were supplemented with 4% paraformaldehyd in PBS for 2h at RT. Medium was discarded and cells were air-dried over night. Samples were fixed with 1:1 mixture of aceton-methanol for 10 min, dried again and stained for CD4 using purified mouse-anti human antibody (clone RPA-T4, BioLegend, Uithoorn, The Netherlands) for 30 min. Samples were then washed with PBS supplemented with 1% BSA and subsequently incubated with Cy3 conjugated goat-anti-mouse Ig (BD Bioscience) and the nuclear dye DAPI (Fluka-SigmaAldrich, Taufkirchen, Germany) for another 30 min. Cells were embedded using Mowiol solution (17% Mowiol in Tris/glycerin buffer) and analyzed using AxioImagerM1 (Zeiss) and AxioVision 4.6 software (Zeiss).

### Identification of fluorescence uptake in vivo

Murine spleen, lymph node (mesenteric and inguinal) and bone-morrow cells were isolated from individual mice. To obtain single-cell suspensions, organs were minced through a 70µm nylon mesh and washed with PBS supplemented with 2% FCS. Red blood cells were lysed in both, bone-marrow cell and splenocyte suspensions. The prepared cells were incubated for 30 min at 4°C with an appropriate combination of different monoclonal antibodies as follows: anti-CD3, anti-CD4, anti-CD8, anti-CD25, anti-NKG2D (BioLegend, Uithoorn, The Netherlands). Dead cells were excluded according to their light-scattering characteristics. All acquisitions were done with a LSRII SORP (BD Biosciences) interfaced to the DIVA software.

### Assessment of transfer of luciferase

Human bulk PBMCs were incubated with CMFDA-labeled luciferase-transgenic L23 cells as described above. After 4h cells were incubated with PE-conjugated anti-CD4 antibodies and T cells which remained negative for green fluorescence were separated from those which revealed cytosol uptake by FACS cell sorting. Isolated CD4^+^ T cells were washed and resuspended in 200µl RPMI medium and transferred to a microtiter plate. 100µg/milliliter luciferin was added to the culture. After 15 min incubation the culture plate was placed in the IVIS chamber (IVIS 200 series, Caliper Life Science, Mainz, Germany) and luminescence was analyzed using Living Image Software (Caliper Life Science, Mainz, Germany). 

### Statistics

The Kaplan-Meier plots for survival were assessed. Survival data were analyzed using the Log-rank (Mantel-Cox) Test, all other data were analyzed with the unpaired Student’s 2-tailed T-test using the GraphPad Prism version 5.00 for Windows, San Diego California, USA: *, difference significant with p≤0.05; **, p≤0.001; ***, p≤0.001; a p-value of less than 0.05 was considered significant. 

## Supporting Information

Figure S1 **A: Scale of magnification in scanning electron microscopy show the double sized tumor cells in comparison to lymphocytes.** This enables a separated analysis in flow cytometry because of specifiable identification of lymphocytes and tumor cells in the forward-sideward-scatter (FSC/SSC) leading to weak contamination of tumor cells in the gate of lymphocytes. **B**: Representative FSC/SSC dot blot of the experiments shown in Figure1 after 4 hours of incubation in Eppendorf tubes. The populations of lymphocytes and tumor cells are still separately detectable.
(TIF)

Figure S2 **A: Cytotoxic activity of purified NK cells assessed by JAM test** [39]. L23 cells were labeled with 5µCi 3H thymidin for 16 hours and exposed to different numbers of NK cells for 4 hours. In this test, the reduction of cpm is linear to lysis of target cells since cytolysis induced DNA fragmentation which resulted in small DNA sections that are not withholded in the filter membrane for radioactive analysis. NK cells revealed pronounced cytotoxicity against L23 cells. B: 2x106 human PBMCs were incubated with 2x106 CMFDA-labeled L23 cells for 4 h, subsequently stained for NK cell marker NKp46 and analyzed in flow cytometry. The exposure of lymphocytes to L23 cells remained NK cells unaffected.
(TIF)

Figure S3 **A: Proliferation analysis of 5x10^4^ T cells sorted in non-fluorescent (CMFDA^-^) and fluorescent (CMFDA^+^) CD4^+^ T cells after 5 days culture without any additional stimulation.** Cells were sorted after 4 h incubation of bulk PBMCs to CMFDA-labeled L23 cells.
**B**: Preincubation of PBMC with OKT3 led to TCR internalization which could be followed by staining with mAb against TCR. **C**: Assessment of cytosol incorporation after treatment of PBMC with 0,3µg/ml OKT3 for 1 hour at 37°C which rather increased the effect than abolished the exchange of cytosol. **D**: PBMC were exposed to CMFDA-labeled L23 cells untreated or treated with blocking MHC II antibodies. L23 cells were incubated 30 min on ice before culture with PBMC. Cells were washed and subsequently added to the lymphocytes. **E**: PBMCs were activated prior exposure to tumor cells specifically with 2x10^3^ irradiated L23 cells or unspecific using 100Uml IL-2 for 3 days. Afterwards naive and activated PBMCs were incubated with labeled L23 for 4 h. The activation state did not affect the interaction with L23 cells and transfer of cytosol.
(TIF)

Figure S4 **A: 2x10^6^ purified mouse CD4^+^ T cells were incubated with 2x10^6^ cells of the murine B lymphoma cell line BM185 for 4 h and prepared for EM.** Scanning (i and ii) and transmission (iii) EM revealed contacts between T cells and tumor cells which caused polarization of the lymphocytes but which were not as intense as could be observed with human T cells and L23 cells. **B**: Just like the human lymphocytes incubated with porcine tumor cells, the populations of splenocytes from BALB/c mice and the BALB/c derived BM185 are distinguishable in the FSC/SSC thus, the populations can be analyzed separately for the uptake of fluorescent cytosol.
(TIF)

Figure S5 **Splenocytes derived from Balb/c and 6.5 mice were exposed to equal numbers of CMFDA-labeled BM185 wt or HA-expressing transgeneic BM185 for 4 h *invitro*.** About 20% of CD4^+^ T cells derived from 6.5 mice express a TCR specific for HA. The uptake of fluorescence was indeed higher with BM185-HA but this accounts for splenocytes from BALB/c as well.
(TIF)
