# Supplementary figures and images for: Exchange of Cytosolic Content between T Cells and Tumor Cells Activates CD4 T Cells and Impedes Cancer Growth
Source: PLoS One. 2013 Oct 24;8(10):e78558. doi: 10.1371/journal.pone.0078558 (PMC3813479; doi:10.1371/journal.pone.0078558)

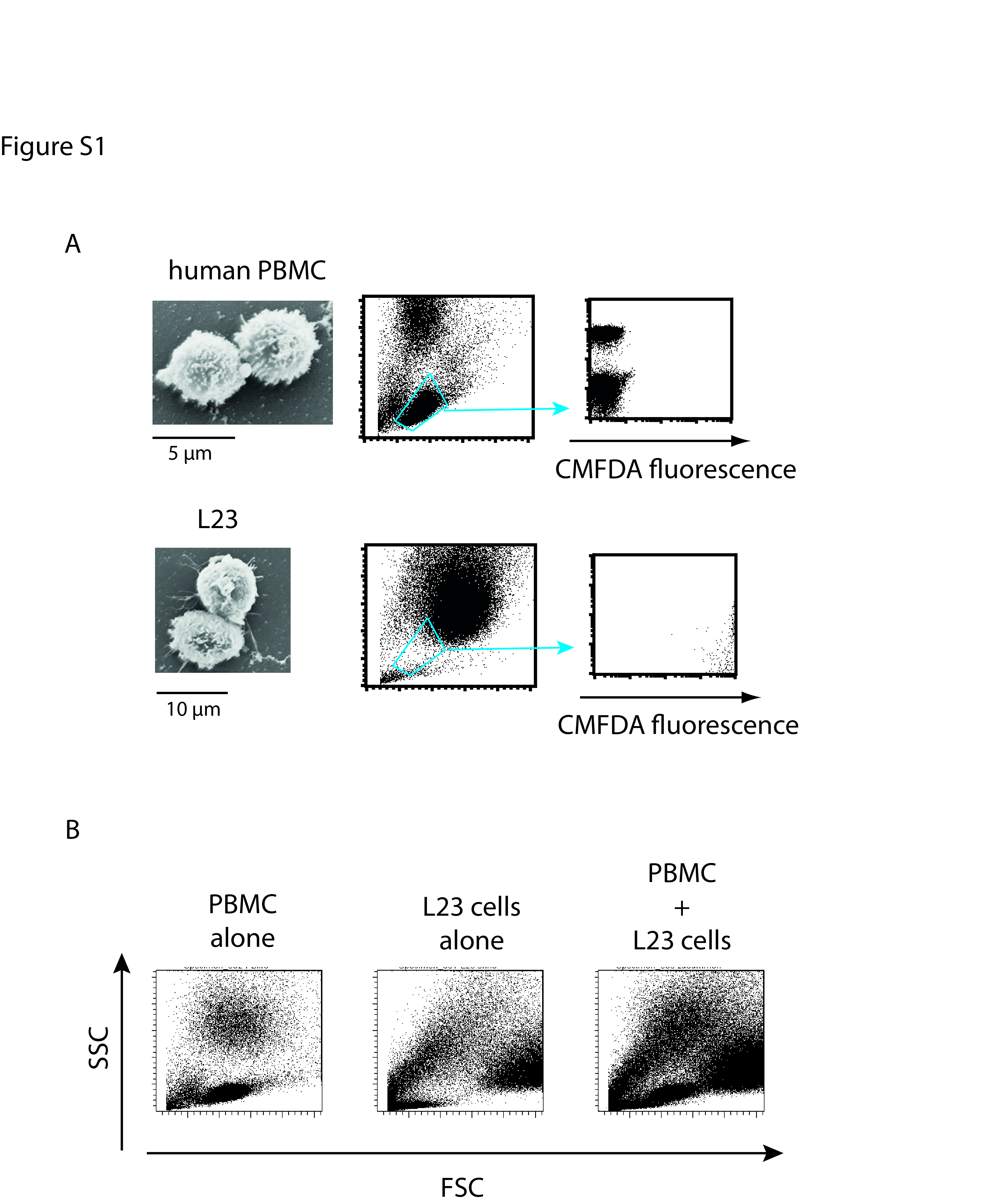

Supplement: Figure S1 — A: Scale of magnification in scanning electron microscopy show the double sized tumor cells in comparison to lymphocytes. This enables a separated analysis in flow cytometry because of specifiable identification of lymphocytes and tumor cells in the forward-sideward-scatter (FSC/SSC) leading to weak contamination of tumor cells in the gate of lymphocytes. B: Representative FSC/SSC dot blot of the experiments shown in Figure1 after 4 hours of incubation in Eppendorf tubes. The populations of lymphocytes and tumor cells are still separately detectable. (TIF) [file pone.0078558.s001.tif]

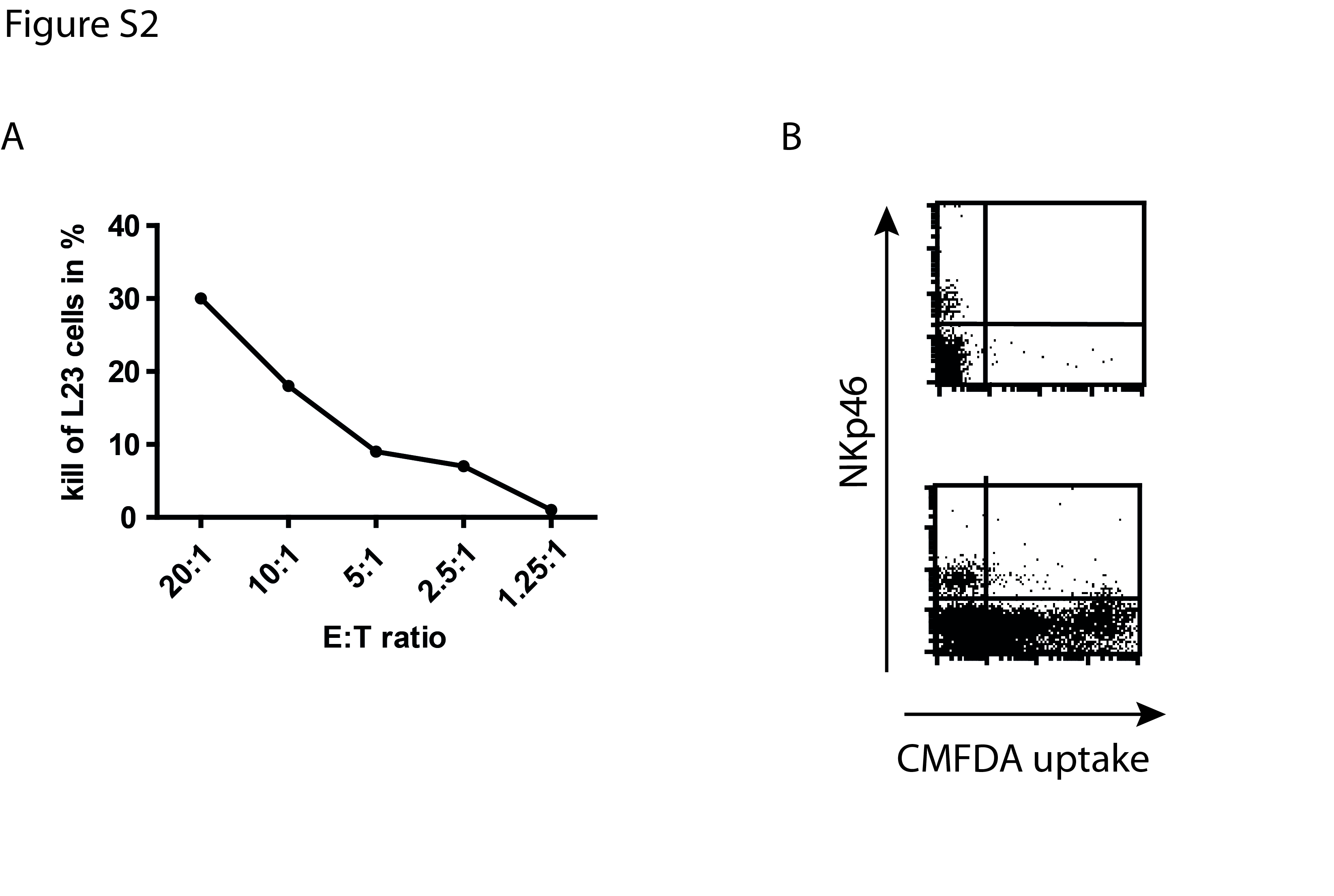

Supplement: Figure S2 — A: Cytotoxic activity of purified NK cells assessed by JAM test [39]. L23 cells were labeled with 5µCi 3H thymidin for 16 hours and exposed to different numbers of NK cells for 4 hours. In this test, the reduction of cpm is linear to lysis of target cells since cytolysis induced DNA fragmentation which resulted in small DNA sections that are not withholded in the filter membrane for radioactive analysis. NK cells revealed pronounced cytotoxicity against L23 cells. B: 2x10^6^ human PBMCs were incubated with 2x10^6^ CMFDA-labeled L23 cells for 4 h, subsequently stained for NK cell marker NKp46 and analyzed in flow cytometry. The exposure of lymphocytes to L23 cells remained NK cells unaffected. (TIF) [file pone.0078558.s002.tif]

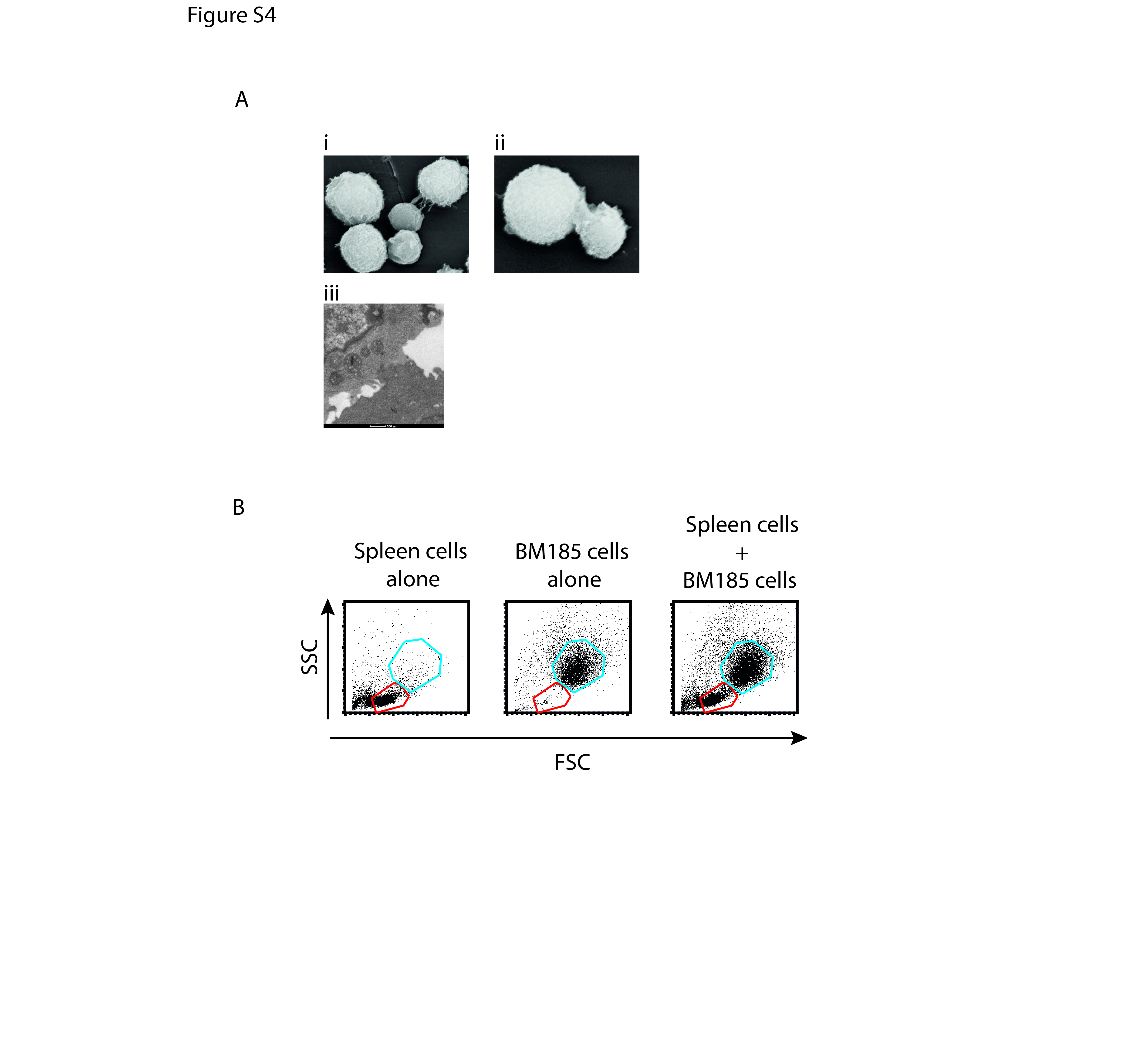

Supplement: Figure S4 — A: 2x10^6^ purified mouse CD4+ T cells were incubated with 2x10^6^ cells of the murine B lymphoma cell line BM185 for 4 h and prepared for EM. Scanning (i and ii) and transmission (iii) EM revealed contacts between T cells and tumor cells which caused polarization of the lymphocytes but which were not as intense as could be observed with human T cells and L23 cells. B: Just like the human lymphocytes incubated with porcine tumor cells, the populations of splenocytes from BALB/c mice and the BALB/c derived BM185 are distinguishable in the FSC/SSC thus, the populations can be analyzed separately for the uptake of fluorescent cytosol. (TIF) [file pone.0078558.s004.tif]

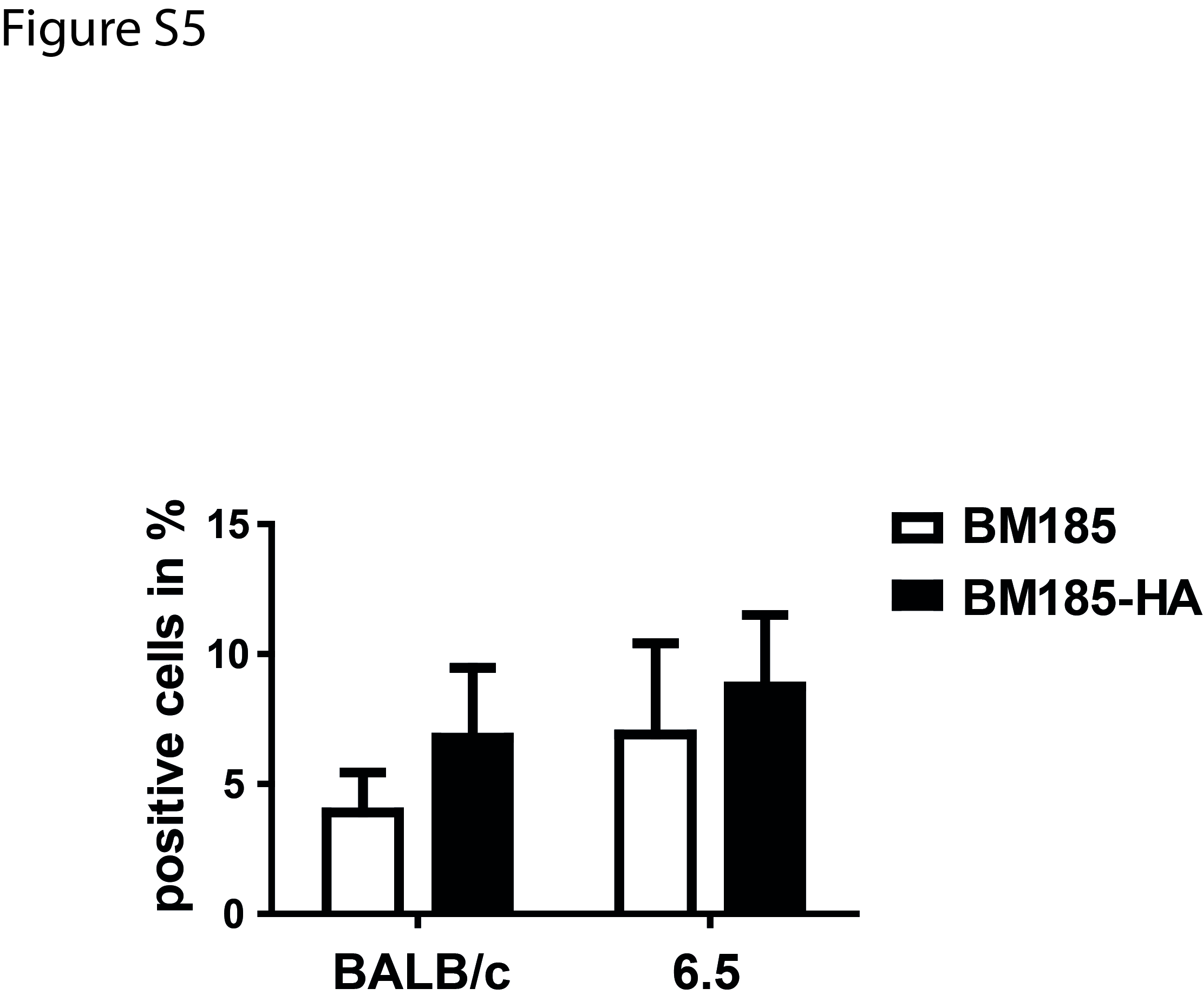

Supplement: Figure S5 — Splenocytes derived from Balb/c and 6.5 mice were exposed to equal numbers of CMFDA-labeled BM185 wt or HA-expressing transgeneic BM185 for 4 h invitro. About 20% of CD4+ T cells derived from 6.5 mice express a TCR specific for HA. The uptake of fluorescence was indeed higher with BM185-HA but this accounts for splenocytes from BALB/c as well. (TIF) [file pone.0078558.s005.tif]
